# Black Phosphorus/MnO_2_ Nanocomposite Disrupting Bacterial Thermotolerance for Efficient Mild‐Temperature Photothermal Therapy

**DOI:** 10.1002/advs.202303911

**Published:** 2023-09-12

**Authors:** Feng Wang, Qinghe Wu, Guoping Jia, Lingchi Kong, Rongtai Zuo, Kai Feng, Mengfei Hou, Yimin Chai, Jia Xu, Chunfu Zhang, Qinglin Kang

**Affiliations:** ^1^ Department of Orthopedics Shanghai Sixth People's Hospital Affiliated to Shanghai Jiao Tong University School of Medicine School of Biomedical Engineering Shanghai Jiao Tong University Shanghai 200030 China

**Keywords:** drug‐resistant bacteria, heat shock protein, photothermal therapy, respiratory chain complex, self‐cascade nanozyme

## Abstract

The emergence of multi‐drug resistant (MDR) pathogens is a major public health concern, posing a substantial global economic burden. Photothermal therapy (PTT) at mild temperature presents a promising alternative to traditional antibiotics due to its biological safety and ability to circumvent drug resistance. However, the efficacy of mild PTT is limited by bacterial thermotolerance. Herein, a nanocomposite, BP@Mn‐NC, comprising black phosphorus nanosheets and a manganese‐based nanozyme (Mn‐NZ) is developed, which possesses both photothermal and catalytic properties. Mn‐NZ imparts glucose oxidase‐ and peroxidase‐like properties to BP@Mn‐NC, generating reactive oxygen species (ROS) that induce lipid peroxidation and malondialdehyde accumulation across the bacterial cell membrane. This process disrupts unprotected respiratory chain complexes exposed on the bacterial cell membrane, leading to a reduction in the intracellular adenosine triphosphate (ATP) content. Consequently, mild PTT mediated by BP@Mn‐NC effectively eliminates MDR infections by specifically impairing bacterial thermotolerance because of the dependence of bacterial heat shock proteins (HSPs) on ATP molecules for their proper functioning. This study paves the way for the development of a novel photothermal strategy to eradicate MDR pathogens, which targets bacterial HSPs through ROS‐mediated inhibition of bacterial respiratory chain activity.

## Graphical abstract

A nanocomposite, BP@Mn‐NC, comprising black phosphorus nanosheets and a manganese‐based nanozyme with photothermal and catalytic properties is developed. BP@Mn‐NC interferes with bacterial respiratory chain complexes and inhibits heat shock proteins (HSPs), leading to antibacterial effects under low‐power irradiation‐based mild photothermal therapy. This specific impairment of bacterial HSPs offers a promising photothermal strategy to eradicate multi‐drug resistant infections.

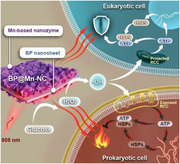

## 1 Introduction

The annual death toll from multi‐drug resistant (MDR) pathogens currently exceeds 700000 and is predicted to rise to 10 million by 2050, surpassing the number of deaths attributed to cancer.^[^ ^1^ ^]^ Gram‐negative *Escherichia coli* (*E. coli*) is a major cause of various infectious diseases and has acquired resistance to a wide range of antibiotics.^[^ ^2^ ^]^ Due to the challenge of developing novel antibiotic agents in the pharmaceutical industry, there is an urgent need to develop non‐drug treatments that can combat MDR *E. coli* infections. Near‐infrared (NIR) irradiation‐based photothermal therapy (PTT) is a promising physical antibacterial technology for avoiding drug resistance and achieving precision medicine.^[^ ^3^, ^4^ ^]^ However, a significant limitation of photothermal effect‐based therapies is the potential damage they may cause to the surrounding healthy cells and tissues, even with accurate targeting of specific area.^[^ ^5^ ^]^ This becomes inevitable when high‐power (> 1 W cm^−2^) irradiation induces local hyperthermia (>50 °C). To overcome this limitation, mild PTT (<45 °C) has garnered more attention in recent years due to its favorable biological safety.^[^ ^6^, ^7^ ^]^ However, the effectiveness of PTT in eliminating bacteria decreases as the heat energy decreases, significantly hindering its clinical application.^[^ ^8^, ^9^ ^]^

To enhance the efficacy of mild PTT against bacterial infections, previous studies have explored methods to increase the specificity of photothermal components for bacteria, such as electrostatic attraction.^[^ ^10^, ^11^ ^]^ However, it is reasonably expected that the subsequent photothermal effect would heat not only the bacteria but also the infected foci and surrounding tissues, posing a challenge in balancing antibacterial efficiency and biological safety. Fortunately, the distinct sensitivity of prokaryotes and eukaryotes to external stimuli offers a potential solution. Unlike eukaryotic cells, where the respiratory chain is safeguarded by the mitochondrial inner membrane,^[^ ^12^ ^]^ bacterial cells expose this essential component for electron transfer and adenosine triphosphate (ATP) production on the cytoplasmic membrane.^[^ ^13^ ^]^ Furthermore, the respiratory chain complexes in bacteria lack certain protective auxiliary subunits and only consist of key functional subunits required for oxidation reactions.^[^ ^14^, ^15^ ^]^ This potentially make bacteria more susceptible to reactive oxygen species (ROS) imbalance and lipid peroxidation (LPO) of the cell membrane compared to eukaryotic cells. Notably, the expression and function of heat shock proteins (HSPs), which are crucial stress proteins for cellular thermal tolerance, rely heavily on ATP molecules.^[^ ^16^, ^17^ ^]^ Therefore, it is plausible to assume that specifically impairing the respiratory chain in bacteria could significantly disrupt their tolerance to hyperthermia without affecting tissue cells. In fact, previous studies have demonstrated the significant antimicrobial efficacy of ROS‐based therapy in combination with PTT.^[^ ^18^, ^19^ ^]^ However, further investigation is required to determine whether this therapeutic strategy can achieve a balance between antibacterial efficacy and biological safety. Moreover, the involvement of respiratory chain complexes and HSPs in its mechanism of action remains unexplored.

Black phosphorus (BP) nanosheets have emerged as a promising 2D nanomaterial due to their inherent advantages, including excellent modifiability, reliable biocompatibility, and desirable biodegradability,^[^ ^20^, ^21^ ^]^ distinguishing them from alternative photothermal agents. Previous studies have demonstrated that BP can induce membrane damage and ROS‐dependent oxidative stress in microorganisms, contributing to its anti‐infection properties,^[^ ^22^, ^23^ ^]^ in addition to its photothermal effect. During photocatalytic generation of ROS, BP nanosheets exhibit a response to visible and ultraviolet light, leading to the generation of singlet oxygen and hydroxyl radicals (·OH), respectively.^[^ ^24^, ^25^ ^]^ However, the penetration of visible and ultraviolet light in tissues is significantly limited.^[^ ^26^ ^]^ Even if BP is modified to respond to NIR light, such as through assembly into nanocomplexes^[^ ^27^ ^]^ or fabrication into quantum dots,^[^ ^28^ ^]^ the dependence on excitation light restricts the generation of ROS to a short period of irradiation exposure. Consequently, it becomes challenging to achieve the optimal synergistic effect for mild PTT. Therefore, it is crucial to develop an improved strategy for BP that enables efficient ROS generation independent of photoexcitation. In a previous study, we successfully developed an ultra‐small manganese‐based nanozyme (Mn‐NZ) that could efficiently generate ROS through a cascade reaction by catalyzing decomposition of glucose.^[^ ^29^, ^30^ ^]^ Due to its ultra‐small particle size, mild reaction conditions, and efficient catalytic activity, Mn‐NZ holds great potential for synergizing with BP‐mediated mild PTT for anti‐infection.

**Scheme 1:**
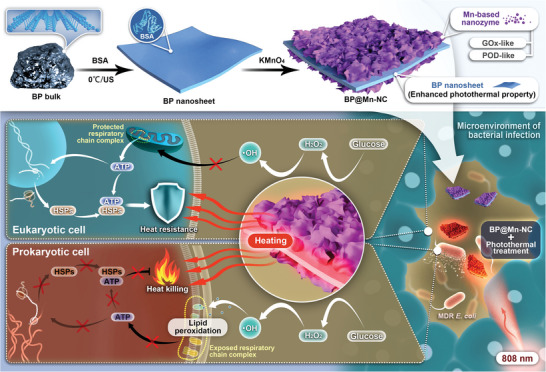
Schematic illustration of the enhanced antibacterial efficacy of mild PTT against MDR *E. coli* by specifically disrupting bacterial thermotolerance using BP@Mn‐NC.

In this study, we first prepared 2D BP nanosheets by ultrasonication‐mediated exfoliation method in the presence of bovine serum albumin (BSA). Subsequently, these nanosheets served as a growth template for Mn‐NZ, culminating in the formation of a nanocomposite, denoted as BP@Mn‐NC. Integration of Mn‐NZ with BP enhanced the photothermal conversion efficiency of BP and endowed it with catalytic activity for spontaneous ROS generation. The treatment of MDR *E. coli* with BP@Mn‐NC resulted in a significant ROS overload, leading to cell membrane LPO and the accumulation of malondialdehyde (MDA), which caused a notable decrease in respiratory chain activity and thus ATP production. Consequently, there was no prompt upregulation of HSPs expression upon irradiation exposure and MDR *E. coli* became vulnerable to mild PTT. Importantly, BP@Mn‐NC exhibited negligible impact on the respiratory chain activity and viability of L929 cells at the antibacterial concentration, indicating its specific antibacterial effect. Ultimately, this approach efficiently eliminated MDR *E. coli* abscesses and demonstrated responsible biocompatibility (**Scheme** **1**). We anticipate that this BP@Mn‐NC‐based antibacterial strategy, which exploits the specific inhibition of bacterial respiratory chain and HSPs by ROS to sensitize bacteria to mild PTT, holds a great potential for clinical translation.

## 2 Results and Discussion

### 2.1 Synthesis and Characterizations of BP@Mn‐NC

**Figure 1:**
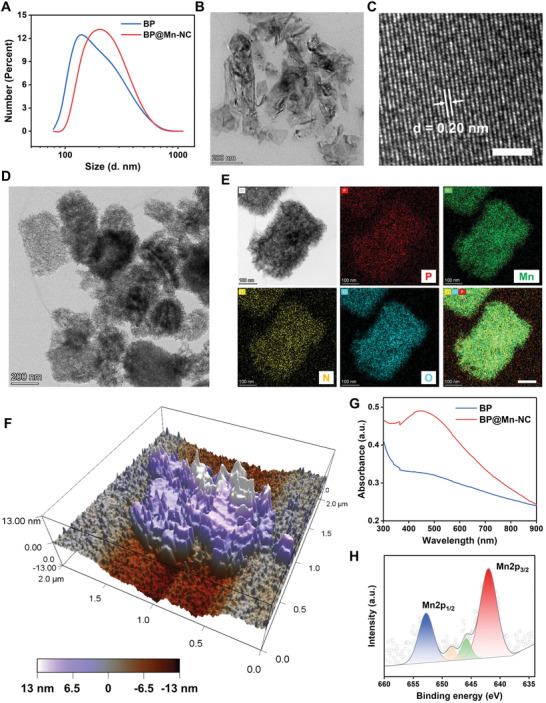
Characterizations of BP and BP@Mn‐NC. A) Hydrodynamic diameter distribution of BP and BP@Mn‐NC. B) TEM image of BP. C) High‐resolution TEM of BP. Scale bar: 2 nm. D) TEM image of BP@Mn‐NC. E) Elemental mapping analysis of BP@Mn‐NC. Scale bar: 100 nm. F) 3D atomic force microscope image of BP@Mn‐NC. G) UV–vis absorption spectra of BP and BP@Mn‐NC. H) Mn2p XPS spectrum of BP@Mn‐NC.

The BP@Mn‐NC was synthesized by two steps, which involved ultrasonication‐mediated exfoliation of BP nanosheets using BSA as a stabilizing agent and subsequent in‐situ growth of Mn‐NZ on the surface of BSA‐stabilized BP through the reduction of KMnO_4_ (Scheme 1). The hydrodynamic diameters of the as‐synthesized BP and BP@Mn‐NC were ≈150 and 200 nm, respectively (**Figure** **1**A). The successful exfoliation of BP with BSA as a stabilizer was confirmed by transmission electron microscopy (TEM). BSA‐stabilized BP had a smooth surface and high crystallinity with the lattice spacing of 0.20 nm (Figure 1B,C), while BP@Mn‐NC exhibited a wrinkled surface (Figure 1D). Elemental mapping and energy‐dispersive spectroscopy (EDS) analysis revealed the presence of phosphorus, manganese, nitrogen, and oxygen in BP@Mn‐NC, indicating the successful integration of BSA‐stabilized BP and Mn‐NZ as a nanocomposite (Figure 1E; Figure S1, Supporting Information). Atomic force microscopy (AFM) analysis revealed that BP had a height of ≈4 nm with a relatively smooth surface (Figure S2, Supporting Information), while BP@Mn‐NC had a height ranging from 4 to 10 nm with numerous small pinnacles attributed to BSA‐directed growth of Mn‐NZ (Figure 1F; Figure S3, Supporting Information). The UV‐vis absorption spectra of BP and BP@Mn‐NC showed broad‐spectrum absorption, with BP@Mn‐NC exhibiting stronger absorption. The X‐ray photoelectron spectroscopy (XPS) analysis of BP@Mn‐NC identified the characteristic binding energy peaks of Mn^4+^ at 2p_3/2_ (642 eV) and 2p_1/2_ (652.8 eV) (Figure 1H; Figure S4, Supporting Information), indicating the presence of MnO_2_ nanoparticles as constituents of Mn‐NZ. These results suggest that the BSA‐mediated exfoliation could synthesize BSA‐coated BP nanosheets, which could act as a template to enable the growth of MnO_2_ nanozyme in the BSA coating layer.

### 2.2 Photothermal and Catalytic Properties of BP@Mn‐NC

**Figure 2:**
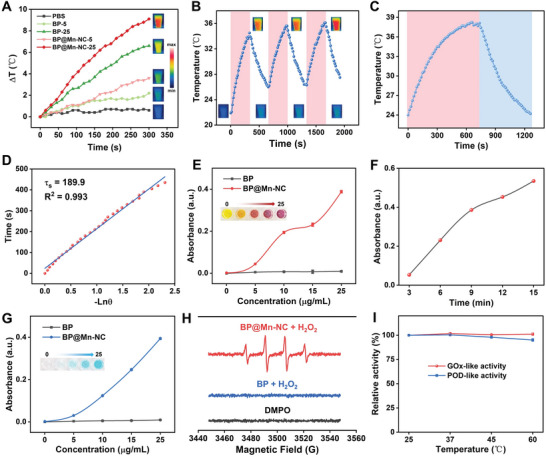
Photothermal effect and enzymatic performance of BP@Mn‐NC. A) Concentration‐dependent photothermal effect of BP and BP@Mn‐NC. B) Photothermal stability of BP@Mn‐NC through three cycles of heating/cooling process. C) Heating‐cooling profile of BP@Mn‐NC for a laser on/off cycle. D) Linear cooling time versus ‐ln(θ) obtained from the cooling period of figure (C). E) Concentration‐dependent GOx‐like activity of BP and BP@Mn‐NC. F) Time‐dependent GOx‐like activity of BP@Mn‐NC. G) Concentration‐dependent POD‐like activity of BP and BP@Mn‐NC. H) ESR analysis of ·OH generated by BP and BP@Mn‐NC using DMPO as the trapping agent. I) Stability of GOx‐ and POD‐like activity of BP@Mn‐NC at different temperatures.

The photothermal effect of BP and BP@Mn‐NC was assessed by irradiating their suspensions with an 808 nm laser at a power density of 0.688 W cm^−2^. Both BP and BP@Mn‐NC exhibited concentration‐ and irradiation time‐dependent temperature elevation; however, BP@Mn‐NC demonstrated a higher increase (9.1 °C) compared to BP (6.6 °C) at a concentration of 25 µg mL^−1^ (based on the mass concentration of BP) after 5 min of irradiation (**Figure** **2**A). This finding indicated that BP@Mn‐NC possessed superior photothermal conversion effect. The photothermal stability of both BP and BP@Mn‐NC was assessed by illuminating their suspensions (25 µg mL^−1^) for a repeated heating/cooling (5 min/5 min) process. After three cycles, the temperature of the suspensions could still reach the initial peak values, indicating their thermal stability (Figure 2B; Figure S5, Supporting Information). To determine the photothermal conversion efficiency (PTCE), BP and BP@Mn‐NC suspensions (25 µg mL^−1^) were irradiated by the laser until the temperature reached a plateau, followed by cooling to the ambient temperature (Figure 2C; Figure S5, Supporting Information). By analyzing the plots of cooling time versus the negative natural logarithm of the driving force temperature, the PTCE of BP and BP@Mn‐NC was calculated to be 42.73% and 60.56%, respectively (Figure 2D; Figure S5, Supporting Information). The ultra‐high PTCE of BP@Mn‐NC makes it a promising candidate for mild PTT at low power density.

The catalytic activities of BP@Mn‐NC, including its glucose oxidase (GOx)‐like and peroxidase (POD)‐like activities, were subsequently evaluated. The GOx‐like activity was assessed using a commercial GOx assay kit that determines the production of hydrogen peroxide through glucose oxidation. The results revealed that BP@Mn‐NC showed concentration‐dependent and time‐increasing GOx‐like activity, while BP exhibited negligible catalytic activity (Figure 2E,F). To assess the POD‐like activity of BP and BP@Mn‐NC, a 3,3′,5,5′‐tetramethylbenzidine (TMB)‐based chromogenic reaction was employed. In this reaction, the POD‐mediated generation of ·OH led to the oxidation of TMB, forming blue‐colored ox‐TMB with absorbance at 650 nm. Compared to BP, BP@Mn‐NC demonstrated a strong concentration‐dependent POD‐like activity (Figure 2G; Figure S6, Supporting Information). Furthermore, the electron spin resonance (ESR) analysis confirmed the ·OH‐generating activity of BP@Mn‐NC, as its ESR spectrum displayed a characteristic four‐peak pattern of ·OH with an intensity ratio of 1:2:2:1, which was not present in BP (Figure 2H). To evaluate the thermal stability of the catalytic activity of BP@Mn‐NC, we incubated BP@Mn‐NC at different temperature conditions and then assessed its catalytic performance. The results showed that both the GOx‐ and POD‐like activities of BP@Mn‐NC were thermally stable, retaining 100% and 95% of their respective activities at 60 °C (Figure 2I; Figure S7, Supporting Information). Overall, the exceptional photothermal conversion and ROS‐generating capability of BP@Mn‐NC highlight its potential for facilitating efficient mild PTT.

### 2.3 In Vitro Antibacterial Activity and Biocompatibility of BP@Mn‐NC

**Figure 3:**
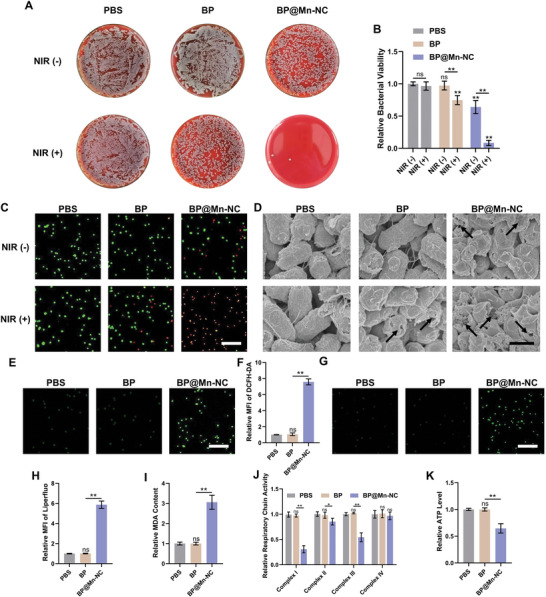
Antibacterial performance of BP@Mn‐NC‐mediated mild PTT. A) Photographs of bacterial colonies and B) viability analysis of MDR *E. coli* treated with BP or BP@Mn‐NC (25 µg mL^−1^) with or without NIR irradiation exposure (808 nm, 0.688 W cm^−2^) (*n* = 3). C) Live/dead fluorescence staining of bacteria after different treatments. Scale bar: 20 µm. D) SEM images of bacteria in different treatment groups for morphological damages detection (black arrow). Scale bar: 1 µm. E) The fluorescence staining and F) quantitative analysis of intracellular ROS in MDR *E. coli* treated with BP or BP@Mn‐NC (25 µg mL^−1^) using DCFH‐DA as the probe (*n* = 3). Scale bar: 20 µm. G) The fluorescence staining and H) quantitative analysis of LPO of MDR *E. coli* after different treatments using Liperfluo as the probe (*n* = 3). Scale bar: 20 µm. I) Intracellular MDA content, J) respiratory chain complex activity, and K) ATP level of bacteria treated by BP or BP@Mn‐NC (25 µg mL^−1^) (n = 3). *P < 0.05, **P < 0.01, ^ns^ *P* > 0.05.

The dual catalytic property of BP@Mn‐NC is expected to enhance the efficacy of subsequent mild PTT by inducing bacterial vulnerability. To investigate this hypothesis, MDR *E. coli* were pretreated with BP or BP@Mn‐NC (25 µg mL^−1^) for 2 h, followed by the NIR irradiation (808 nm, 0.688 W cm^−2^) for 3 min, then a blood agar plate assay was first conducted to evaluate the antibacterial effect of BP@Mn‐NC‐based mild PTT against MDR *E. coli*. The results demonstrated that BP alone did not exhibit significant antibacterial activity. In contrast, BP@Mn‐NC exhibited mild inhibition of bacterial proliferation (**Figure** **3**A,B). Additionally, BP‐mediated mild PTT upon NIR irradiation displayed a moderate antibacterial efficacy (Figure 3A,B), consistent with previous studies.^[^ ^31^ ^]^ Notably, MDR *E. coli* pretreated with BP@Mn‐NC lost its ability to form colonies on blood agar plates after laser exposure (Figure 3A,B), revealing the superior antibacterial efficacy of BP@Mn‐NC‐mediated mild PTT. To further verify our findings, a bacterial live/dead staining with SYTO9 and PI probes was conducted using the treated MDR *E. coli*. The results indicated that BP@Mn‐NC‐mediated PTT effectively killed MDR *E. coli*, as shown by the strong red fluorescence of the PI probe (Figure 3C), which indicated the dead bacteria.^[^ ^32^ ^]^ However, regardless of the treatment with BP@Mn‐NC alone or BP in combination with NIR irradiation exposure, the dominant fluorescence observed in bacteria was still green (Figure 3C), suggesting that bacterial activity was only mildly impaired. In addition to the blood agar plate assay and live/dead staining, scanning electron microscopy (SEM) was also utilized to observe the bacterial morphological changes after the treatment of mild PTT mediated by BP or BP@Mn‐NC. The results showed that MDR *E. coli* treated with BP@Mn‐NC exhibited pronounced damages, while bacteria under mild PTT mediated by BP only showed slight deformation compared to the normal morphology of fimbriae and capsules (Figure 3D). Furthermore, NIR laser irradiation further strengthened the bactericidal effect of BP@Mn‐NC, causing severe morphological changes such as surface collapse and fracture (Figure 3D). These results indicate that pretreatment with BP@Mn‐NC significantly enhances the sensitivity of bacteria to mild PTT and demonstrate the potential of BP@Mn‐NC‐mediated PTT for effectively killing MDR *E. coli*, supporting its candidacy as an alternative antibacterial treatment strategy.

The enhanced efficacy of BP@Mn‐NC‐mediated mild PTT could potentially be attributed to the metabolic abnormalities induced by ROS generated from the dual catalytic activity of BP@Mn‐NC. Therefore, MDR *E. coli* were incubated with BP or BP@Mn‐NC (25 µg mL^−1^) for 2 h and the intracellular ROS content and LPO level were then measured using DCFH‐DA (10 µm) and Liperfluo (1 µm) probes, respectively. The results showed that BP@Mn‐NC significantly increased ROS accumulation and caused substantial LPO in bacterial cells, as evidenced by the intense green fluorescence of the DCFH‐DA and Liperfluo. (Figure 3E–H). Conversely, BP treatment did not result in significant changes in intracellular ROS and LPO levels (Figure 3E–H), suggesting the effectiveness of dual catalytic properties of BP@Mn‐NC in generating bactericidal ROS. Moreover, the content of MDA, an essential metabolite of LPO, was measured in the BP‐ or BP@Mn‐NC‐treated MDR *E. coli*. Consistent with the ROS and LPO measurement results, BP@Mn‐NC treatment significantly promoted intracellular MDA accumulation, whereas BP treatment did not yield similar results (Figure 3I). The impact of BP@Mn‐NC on the bacterial morphology visualized by SEM (Figure 3D) could be attributed to the exacerbation of damage to the phospholipid bilayer caused by MDA.^[^ ^33^ ^]^ In addition, MDA has been found to affect the activity of respiratory chain complexes in vitro.^[^ ^34^, ^35^ ^]^ Therefore, bacterial respiratory chain activity is expected to be impaired upon BP@Mn‐NC treatment directly through ROS‐mediated MDA accumulation, leading to the inhibition of ATP synthesis. To test this hypothesis, the activities of respiratory chain complex I‐IV and ATP content were examined in MDR *E. coli* after 2 h of incubation with BP or BP@Mn‐NC (25 µg mL^−1^). The results revealed that BP@Mn‐NC treatment led to the inhibition of respiratory chain complexes I, II, and III, subsequently resulting in a decrease in the intracellular ATP content of MDR *E. coli* (Figure 3J,K).

**Figure 4:**
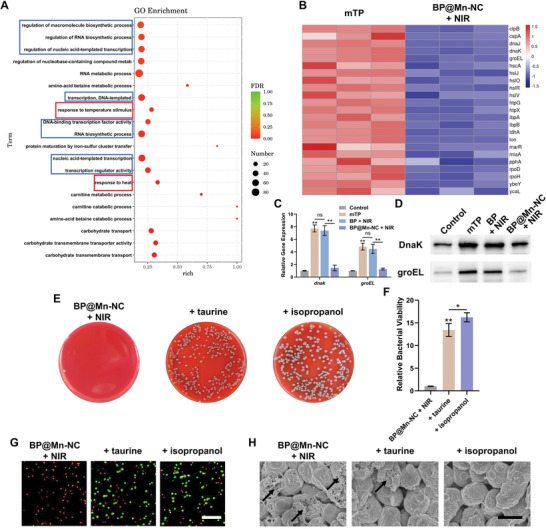
Disrupted metabolic processes of MDR *E. coli* treated by BP@Mn‐NC‐mediated mild PTT. A) GO enrichment of downregulated genes in MDR *E. coli* treated by BP@Mn‐NC‐mediated (25 µg mL^−1^) mild PTT (808 nm, 0.688 W cm^−2^) compared to bacteria treated by mild thermal processing (mTP; 45°C, 3 min). B) Heatmap of differentially expressed genes involved in bacterial thermotolerance of MDR *E. coli* in mTP and BP@Mn‐NC + NIR groups. C) Relative gene expression of bacterial heat shock proteins *DnaK* and *groEL* of MDR *E. coli* treated by mTP or BP‐ or BP@Mn‐NC‐mediated mild PTT (*n* = 3). D) Representative immunoblots of bacterial Dnak and groEL proteins in different treatment groups. E) Photographs of bacterial colonies and F) viability analysis of bacteria treated by BP@Mn‐NC (25 µg mL^−1^), in supplement with MDA scavenger (taurine, 10 mm) or ·OH scavenger (isopropanol, 1% w/v), followed by NIR irradiation (808 nm, 0.688 W cm^−2^) (*n* = 3). G) Live/dead fluorescence staining of bacteria in different treatment groups. Scale bar: 20 µm. H) SEM images of bacteria in different treatment groups for morphological damages detection (black arrow). Scale bar: 1 µm. *p < 0.05, **p < 0.01.

ATP serves as the primary energy source in living organisms, and ATP deficiency significantly impacts bacterial metabolic processes.^[^ ^36^, ^37^ ^]^ Additionally, previous studies have indicated that the ability of HSPs to help cells withstand external damage, such as hyperthermia, directly relies on ATP molecules.^[^ ^38^ ^]^ To further investigate the mechanism behind the notable enhancement of bacterial sensitivity to mild PTT mediated by BP@Mn‐NC, we conducted RNA‐seq analysis to examine the differentially expressed genes in MDR *E. coli* treated by BP@Mn‐NC (25 µg mL^−1^) along with laser exposure (808 nm, 0.688 W cm^−2^) for mild PTT. MDR *E. coli* subjected to mild thermal processing (mTP) using water bath (45 °C, 3 min) served as the control group. The results revealed that BP@Mn‐NC + NIR treatment influenced the expression of approximately one‐third of bacterial genes compared to mTP (Figure S8, Supporting Information). Specifically, various metabolic processes were significantly disrupted, including response to temperature stimulus and response to heat (**Figure** **4**A). In particular, multiple genes encoding bacterial HSPs failed to be upregulated during BP@Mn‐NC‐mediated mild PTT compared to mTP (Figure 4B). Among these HSPs, clpB (HSP100) exhibits ATPase activity, facilitating the coordinated repair of denatured proteins; ibpA and ibpB (small HSPs) have been reported to inhibit heat‐induced protein aggregation; DnaK (HSP70) serves as a central organizer within the chaperone network and acts upstream of the groEL (HSP60) chaperonin, which provides a cage‐like compartment for the folding of individual protein molecules, ensuring their proper folding without being hindered by aggregation.^[^ ^39^, ^40^ ^]^ The disruption on HSPs explains the vulnerability of BP@Mn‐NC‐treated bacteria to mild PTT. To verify these findings, the expression of two representative bacterial HSPs, DnaK, and groEL, was further examined through quantitative PCR and Western blotting in MDR *E. coli* treated by mTP or mild PTT mediated by BP or BP@Mn‐NC (25 µg mL^−1^). As expected, the expression levels of DnaK and groEL increased rapidly during mTP and BP‐mediated mild PTT (Figure 4C,D). However, during BP@Mn‐NC‐mediated mild PTT, MDR *E. coli* could not upregulate the expression of DnaK and groEL at both the transcriptional and translational levels (Figure 4C,D), indicating impaired thermotolerance. Additionally, RNA‐seq analysis revealed that BP@Mn‐NC + NIR treatment significantly suppressed the transcriptional activity of MDR *E. coli* (Figure 4A; Figure S9, Supporting Information), which may be related to ATP depletion and partially explains the inhibition of HSPs expression. These findings provide an experimental basis for a deeper understanding of the underlying mechanism of BP@Mn‐NC‐mediated mild PTT.

To further investigate the antibacterial effect of BP@Mn‐NC and the roles of ROS and MDA in this process, the MDA scavenger taurine^[^ ^41^ ^]^ (10 mm) and the ·OH scavenger isopropanol^[^ ^42^ ^]^ (1% w/v) were administrated alongside BP@Mn‐NC pretreatment (25 µg mL^−1^) for 2 h, followed by NIR laser exposure (808 nm, 0.688 W cm^−2^), to partially mitigate the effects of MDA and ·OH. The treated bacteria in each group were subjected to the in vitro antibacterial experiments, and the results revealed that taurine substantially diminished the efficacy of BP@Mn‐NC‐mediated mild PTT, as evidenced by the presence of denser bacterial colonies in the plate assay (Figure 4E,F), stronger green fluorescence in the live/dead staining assay (Figure 4G), and less bacterial morphology damages observed under SEM (Figure 4H). Similarly, the ·OH scavenger significantly inhibited the antibacterial function of mild PTT based on BP@Mn‐NC, with the rescue effect being even more pronounced than that of taurine (Figure 4F–H), possibly due to ROS generation occurring upstream in the antibacterial mechanism of BP@Mn‐NC. To assess whether BP@Mn‐NC has a similar cytotoxic effect on the eukaryotic cell, L929 cells, a mouse fibroblast commonly used for cytotoxicity tests, were incubated with BP@Mn‐NC at the concentration of 25 µg mL^−1^ for 2 h before the examination on the respiratory chain activity. As a result, BP@Mn‐NC did not substantially affect the respiratory chain activity of L929 cells (Figure S10, Supporting Information). Furthermore, live/dead staining and a CCK‐8 experiment, based on the reduction of WST‐8 by dehydrogenase in mitochondria, were performed on L929 cells for three consecutive days following mild PTT mediated by BP@Mn‐NC (25 µg mL^−1^). The results demonstrated that L929 cell viability remained unimpaired (Figure S11, Supporting Information), implying their maintained mitochondrial functionality. These results could be attributed to the sophisticated antioxidant protection mechanism^[^ ^43^ ^]^ and the preserved activity of the respiratory chain complex, guaranteed by mitochondria and accessory subunits, in eukaryotic cells compared to bacteria,^[^ ^13^ ^]^ which ensures the targeted action of BP@Mn‐NC on bacteria from a biological perspective. These findings not only confirm the ability of BP@Mn‐NC to generate ROS through dual‐catalytic activity but also demonstrate the advantages of the antibacterial strategy that combines ROS generation and mild PTT, utilizing respiratory chain complexes and HSPs as the bridge, in terms of treatment efficiency and biocompatibility.

### 2.4 Treatment of MDR *E. coli*‐Infected Abscess using BP@Mn‐NC In Vivo

**Figure 5:**
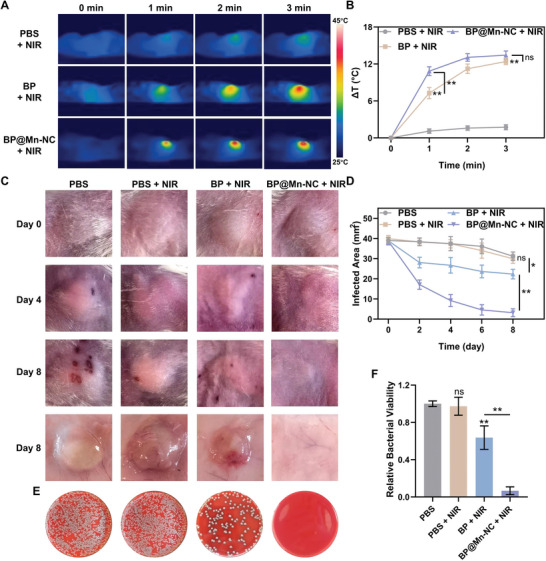
In vivo antibacterial performance of BP@Mn‐NC‐mediated mild PTT. A) Infrared thermal imaging and B) temperature variation of bacterial abscesses treated by PBS, BP (50 µL, 25 µg mL^−1^), or BP@Mn‐NC (50 µL, 25 µg mL^−1^) followed by NIR irradiation exposure (808 nm, 0.8 W cm^−2^) (*n* = 6). C) Photographs and D) quantitative analysis of the abscess areas from infected mice in different treatment groups during treatment period (*n* = 6). E) Photographs of bacterial colonies and F) viability analysis of MDR *E. coli* from the homogenates of infected tissues collected seven days after treatments in different groups (*n* = 3). *p < 0.05, **p < 0.01, ^ns^ *p* > 0.05.

To investigate the potential of BP@Mn‐NC‐mediated antibacterial mild PTT for in vivo application, an abscess animal model was established by subcutaneously injecting a bacterial suspension into the back of BALB/c mice. After 24 h, suspensions of BP or BP@Mn‐NC (50 µL, 25 µg mL^−1^) were inoculated into the abscesses, with an equal volume of PBS solution as the control. Two hours later, the NIR irradiation (808 nm, 0.8 W cm^−2^) was applied and the local temperature was monitored using a thermal imaging camera. The results showed that after a 3 min laser irradiation period, the local temperature in the PBS + NIR group did not increase by >2 °C. However, in the BP + NIR and BP@Mn‐NC + NIR groups, the temperature in the infected area rapidly reached ≈45 °C. Moreover, the local temperature in the BP@Mn‐NC + NIR group increased more rapidly during the initial stage of irradiation (**Figure** **5**A,B), confirming the stronger photothermal conversion efficiency of BP@Mn‐NC compared to BP, which is consistent with the previous in vitro detection results. Eight days after bacterial inoculation, significant abscess formation and skin ulceration occurred in the infected area of PBS and PBS + NIR groups (Figure 5C). The combination of BP and NIR laser irradiation reduced the size of the infection area, although abscess formation was still evident (Figure 5C,D; Figure S12, Supporting Information). In contrast, the BP@Mn‐NC + NIR group exhibited minimal abscess formation, and no local skin was burnt on the irradiation site (Figure 5C,D; Figure S12, Supporting Information). These results indicate that BP@Mn‐NC‐mediated mild PTT is more effective in eliminating MDR *E. coli* infection than BP, while maintaining reliable biocompatibility. Moreover, the infected tissues were harvested seven days after the treatments and homogenized for the blood agar plate assay. The results demonstrated that the BP@Mn‐NC + NIR group had minimal bacterial colony formation, whereas the BP + NIR group exhibited some colony formation, albeit significantly less than the control groups (Figure 5E,F). This finding further supports the superior in vivo antibacterial efficiency of BP@Mn‐NC‐mediated mild PTT.

**Figure 6:**
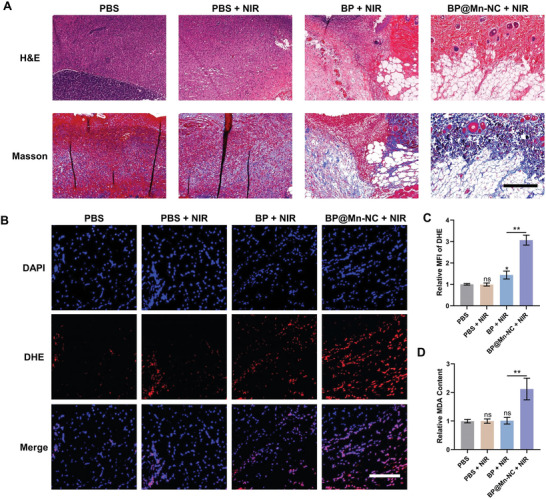
Histological analysis of infected tissues after mild PTT mediated by BP@Mn‐NC. A) H&E and Masson staining of the abscesses seven days after the treatments of PBS, BP (50 µL, 25 µg mL^−1^), or BP@Mn‐NC (50 µL, 25 µg mL^−1^) followed by NIR irradiation exposure (808 nm, 0.8 W cm^−2^). Scale bar: 200 µm. B) Fluorescence staining and C) quantitative analysis of ROS in infected tissues from different treatment groups determined on the second day after treatments (*n* = 3). Scale bar: 300 µm. D) Local MDA content in infected tissues from different treatment groups determined on the second day after treatments using the tissue homogenates (*n* = 3). *p < 0.05, **p < 0.01, ^ns^ *p* > 0.05.

Eight days after bacterial inoculation, the infected tissues were collected for histological examination. The results of H&E and Masson staining revealed the presence of abscesses and necrotic tissues in the infected areas of mice treated with PBS, with or without NIR irradiation (**Figure** **6**A). Although the combination of BP injection and NIR laser irradiation inhibited the infection, abscess formation still occurred (Figure 6A). In contrast, the local tissue of the BP@Mn‐NC + NIR treatment group exhibited distinct normal skin microstructures without any signs of infection or thermal damage, which is consistent with the previous results (Figure 6A). Additionally, blood samples were collected from the mice in the PBS group and BP@Mn‐NC + NIR treatment group two and seven days after the treatment for blood biochemistry analysis, and major organs, including the heart, liver, spleen, lungs, kidneys, and brain, were collected seven days after the treatments and subjected to H&E staining. Consequently, no abnormal fluctuations in major blood biochemical indexes or pathological changes in major organs were observed (Figure S13,A,B, Supporting Information), indicating the biosafety of BP@Mn‐NC‐mediated mild PTT. The results of the hemolysis experiment similarly demonstrated that increasing the concentration of BP@Mn‐NC to 40 µg mL^−1^ did not induce hemolysis (Figure S13C, Supporting Information), further confirming the biosafety of BP@Mn‐NC. Since ROS generation and MDA accumulation played a significant role in the antibacterial effect of BP@Mn‐NC, ROS levels in the infected area were detected two days after different treatments using DHE as the probe. The results showed a slight elevation in ROS levels in the BP + NIR group (Figure 6B,C), which could be attributed to local tissue stress response to mild stimulation of PTT. In contrast, ROS levels in the BP@Mn‐NC + NIR group exhibited a significant increase, as indicated by strong red fluorescence emitted by the DHE probe (Figure 6B,C). Moreover, the local tissues of mice collected two days after treatments were homogenized for the evaluation of MDA content, and the results demonstrated an increase in MDA content in the BP@Mn‐NC + NIR treatment group (Figure 6D). These findings confirm the effectiveness and biosafety of the proposed antibacterial strategy, which utilizes laser‐free ROS generation to synergize mild PTT.

## 3 Conclusion

In summary, we have successfully developed 2D BP@Mn‐NC as an effective nanozyme and PTT agent for efficient and safe antibacterial therapy. During the preparation of BP, BSA molecules were selected as the stabilizer, which also acted as a reducing agent and growth template to introduce the catalytic properties of Mn‐NZ into BP and enhance the photothermal conversion efficiency. BP@Mn‐NC enables irradiation‐free generation of ROS, effectively interfering with exposed and unprotected bacterial respiratory chain complexes and inhibiting HSPs. Subsequently, low‐power irradiation‐based mild PTT exhibited unprecedented antibacterial effects and reliable cytocompatibility. In vivo results demonstrated that BP@Mn‐NC‐mediated mild PTT efficiently eliminated bacterial abscesses while maintaining biosafety. These findings indicate promising clinical translation prospects for the designed BP@Mn‐NC. Furthermore, our work provides a novel perspective for establishing a targeted antibacterial strategy that exploits the specific inhibitory effect of ROS on bacterial respiratory chain and HSPs to sensitize bacteria for mild PTT.

## 4 Experimental Section

### Synthesis and Characterizations of BP@Mn‐NC

BP nanosheets were synthesized using a BSA‐mediated liquid exfoliation method. Initially, 25 mg of bulk BP was mixed with 250 mg of BSA in 50 mL of deoxygenated water and sonicated in the ice water bath for 12 h. The resulting mixture was then centrifuged (3,000 rpm, 5 min) to remove the unexfoliated BP. These nanosheets were further purified through centrifugation at 10,000 rpm for 10 min. Subsequently, KMnO_4_ (1 mg mL^−1^, 100 µL) was dropwise added into the BP nanosheets (1 mg mL^−1^, 500 µL) and reacted for 30 min. The BP@Mn‐NC was collected by centrifugation at 10,000 rpm for 10 min to discard any unreacted KMnO_4_.

Dynamic light scattering data was recorded using a Zetasizer Nano‐ZSP (Malvern, UK). The TEM images and EDS data were acquired on Talos F200X (FEI, USA). AFM images were obtained from MFP‐3D (USA). UV–vis absorption spectrum was recorded using a UV1901PC spectrophotometer (AuCy Instrument, China). X‐ray photoelectron spectroscopy was performed using a Thermo Kalpha spectrometer. Confocal laser scanning microscopy was conducted on TCS SP5 (Leica, Germany).

### Photothermal Effect and Catalytic Properties of BP@Mn‐NC

To examine the concentration‐dependent photothermal effect of BP and BP@Mn‐NC, an 808‐nm laser at 0.688 W cm^−2^ was applied to irradiate BP and BP@Mn‐NC with different concentrations (0, 5, 25 µg mL^−1^; presented by the mass concentration of BP) for 5 min. The heating process was monitored using a thermal infrared camera (FOTRIC, China). The photothermal conversion stability was assessed by irradiating BP (25 µg mL^−1^) or BP@Mn‐NC (25 µg mL^−1^) for three cycles of heating/cooling (5 min/5 min) process.

To determine the PTCE, BP or BP@Mn‐NC (25 µg mL^−1^) was irradiated for heating to reach a steady temperature and then allowed to cool down after turning off the laser. The temperatures of the heating/cooling period were recorded and the PTCE was calculated according to Roper's report.^[^ ^44^ ^]^ The calculation was conducted as following equation:

(1) $$\eta_{T}=\frac{hA\;\left(T_{\max}-T_{\mathrm{amb}}\right)-Q_{0}}{I\;\left(1-10^{-A_{808}}\right)}$$

where *h* represents the heat transfer coefficient, A denotes the surface area of the container, *T*_max_ indicates the equilibrium temperature achieved during laser irradiation, *T*_amb_ refers to the ambient temperature, Q_0_ represents the heat from light absorbed by the cuvette sample walls, I represents the incident laser power (688 mW), and A_808_ corresponds to the absorbance of BP@Mn‐NC (0.386) at 808 nm.

To calculate *h*A, θ and τ_s_ are introduced as the following equations:

(2) $$\theta =\frac{T-T_{\mathrm{amb}}}{T_{\max}-T_{\mathrm{amb}}}$$

(3) $$\tau_{s}=\frac{\sum_{i}m_{i}C_{P,i}}{hA}$$

(4) $$t=-\tau_{s}\ln\left(\theta \right)$$

So, *h*A can be calculated to be mC/τ_s_, where m is the weight of ultrapure water (1 mL) and C is the specific heat capacity of water (4.2 J g^−1^ K). τ_s_ was determined according to the linear curve of cooling time versus ‐ln(θ).

The GOx‐like activity of BP@Mn‐NC was assessed using a glucose oxidase activity assay kit (KTB1310, Abbkine). Briefly, BP@Mn‐NC was first diluted with PBS into different concentrations (0, 5, 10, 15, 25 µg mL^−1^), followed by the mixing of the samples with glucose and chromogen solution according to the vendor's instructions. The absorbance at 580 nm was then measured after incubating for different time periods.

The POD‐like activity of BP@Mn‐NC was tested using a TMB single‐component substrate solution (P0292, Beyotime). Typically, BP@Mn‐NC was first diluted with PBS into different concentrations (0, 5, 10, 15, and 25 µg mL^−1^), followed by the mixing of the samples with the TMB assay solution. The absorbance at 650 nm was recorded after incubating for different time periods. ESR spectroscopy was used to further verify the ·OH generation ability of BP@Mn‐NC. Specifically, BP or BP@Mn‐NC (25 µg mL^−1^) was first incubated with H_2_O_2_ (100 µm) for 3 min, followed by the addition of DMPO (100 mm). The mixture was then transferred into capillary tubes for ESR measurements at G modulation amplitude and 100 G sweep width (Bruker EMX plus, Germany).

The stability of GOx‐ and POD‐like activities of BP@Mn‐NC was evaluated by first incubating BP@Mn‐NC (25 µg mL^−1^) at different temperatures (25, 37, 45, and 60 °C) for 30 min, followed by the activity test according to the method as‐illustrated above.

### In Vitro Antibacterial Activity of BP@Mn‐NC

MDR *E. coli* (Rosetta‐gami2 (DE3)) were cultured in Trypticase Soy Broth Medium (LA0020, Solarbio) at 37 °C for further examination on the antibacterial effect of BP@Mn‐NC. After 12 h of amplification and 3 times of wash with PBS, bacterial suspensions were inoculated into a 96‐well cell culture plate and were treated by BP (25 µg mL^−1^) or BP@Mn‐NC (25 µg mL^−1^) for 2 h at 37 °C in the supplement of glucose (5.5 mm). Bacteria treated with PBS served as the control group. In an alternative experimental sequence, taurine (10 mm) and isopropanol (1% w/v) were administrated alongside BP@Mn‐NC treatment to scavenge malondialdehyde and ·OH, respectively. Then the treated bacteria were irradiated with 808 nm laser exposure (0.688 W cm^−2^) for 3 min, or left without irradiation. After a 10^4^‐fold dilution, 100 µL of bacteria suspension was introduced onto a blood agar plate (B530120, Sangon Biotech) and incubated at 37 °C for 24 h. Thereafter, the colony‐forming units (CFUs) were counted using ImageJ software (National Institutes of Health, Germany), with CFUs per unit volume calculated considering the dilution factor, facilitating the evaluation of relative bacterial viability among the groups. For live/dead assay (L7007, Thermo Fisher Scientific) of MDR *E. coli*, SYTO 9 (1 µL, 6 µm) and PI (1 µL, 30 µm) were added to 50 µL of treated bacteria suspensions, and the mixtures were incubated for 15 min protected from light. Afterward, the stained bacteria were washed and imaged by the confocal laser scanning microscope (Leica, Germany), with the exciting lights set at 488 nm and 561 nm, respectively. For bacterial morphology observation, treated bacteria were fixed with glutaraldehyde (2.5% w/v), dehydrated with gradient ethanol solutions, and subsequently observed using SEM (Hitachi, Japan) after supercritical drying and gold sputter‐coating.

### Intracellular ROS and LPO Detection

Intracellular ROS and LPO levels in MDR *E. coli* stimulated by BP@Mn‐NC were detected using an oxidant‐sensitive fluorescence probe DCFH‐DA (S0033S, Beyotime) and a perylene derivative containing oligooxyethylene Liperfluo (L248, Dojindo), respectively. After 2 h of incubation with BP (25 µg mL^−1^) or BP@Mn‐NC (25 µg mL^−1^) in the supplement of glucose (5.5 mm), the bacteria were stained with DCFH‐DA (10 µm) or Liperfluo (1 µm) for 30 min at 37 °C and washed to remove dissociative probes. Afterward, the bacteria suspensions were observed using a confocal laser scanning microscope and detected using a fluorescence spectrophotometer with the exciting wavelength at 488 nm.

### MDA Level Measurement

To determine the generation of MDA as the metabolite of lipid peroxidation of MDR *E. coli*, an MDA Content Assay Kit (BC0025, Solarbio) was utilized following the instructions provided by the manufacturer. Briefly, BP@Mn‐NC‐treated (25 µg mL^−1^) bacteria were centrifugated at 3600 rpm for 5 min and the obtained bacterial precipitates were mixed with an extracting solution (1 mL) for ultrasonication (200 W). After centrifugation at 8,000 g at 4 °C for 10 min, the supernatants (100 µL) were incubated with the test solution (300 µL) at 100 °C for 60 min and centrifugated at 10 000 g for 10 min. The OD value of the supernatants at 532 and 600 nm were monitored using a microplate reader, facilitating calculating the relative MDA content.

### Bacterial Respiratory Chain Complexes Activities and ATP Content Determination

For evaluating the impairment of the respiratory chain of MDR *E. coli* caused by BP@Mn‐NC treatment, a series of Respiratory Chain Complex Activity Assay Kits (BC0515, BC3235, BC3245, BC0945, Solarbio) were applied according to the manufacturer's instruction. After the bacteria were treated by BP (25 µg mL^−1^) or BP@Mn‐NC (25 µg mL^−1^) for 2 h, the bacteria were centrifugated and the obtained bacterial precipitates were mixed with an extracting solution (1 mL) for homogenate and subsequent centrifugation at 11 000 g for 15 min. Afterward, the precipitates were ultrasonicated (200 W) in extracting solution to prepare the samples for subsequent examinations on the activities of respiratory chain complex I, II, III, and IV using respective detection reagents. The respiratory chain activity of L929 cells was similarly examined after being treated by BP@Mn‐NC (25 µg mL^−1^) for 2 h. Moreover, an ATP Content Assay Kit (BC0300, Solarbio) was applied to evaluate the variation of bacterial ATP content that associated with the activity of respiratory chain complexes. The treated bacteria were ultrasonicated (200 W) for 1 min and centrifuged at 10 000 g for 10 min at 4 °C to obtain the supernatant samples. Then the samples were mixed with chloroform (500 µL) and centrifuged at 10 000 g for 3 min, the obtained supernatant was subjected to the measurement of OD value at 340 nm using the testing reagents and a microplate reader (Thermo Fisher Scientific, USA).

### Cytocompatibility of BP@Mn‐NC

The cytocompatibility of BP@Mn‐NC was determined using the live/dead staining and CCK‐8 assay. Briefly, L929 cells were inoculated in a 96‐well cell culture plate (5000 cells/well). Upon cell adherence, the complete medium was replaced with Dulbecco's modified Eagle's medium supplemented with the antibacterial concentration of BP@Mn‐NC (25 µg mL^−1^) for 2 h of pretreatment followed by 808 nm irradiation exposure (0.688 W cm^−2^) for 3 min. Then the L929 cells were further cultured for 24, 48, and 72 h before the staining with a Calcein‐AM/PI Double Stain Kit (40747ES76, Yeasen). For CCK‐8 assay, treated cells were incubated with 10% CCK‐8 reagent (CK04, Dojindo) for another 2 h after culture. The OD values were measured at 450 nm using a microplate reader to represent the viability of L929 cells.

### RNA Sequencing for Transcriptome Analysis

To comprehensively evaluate the influence of BP@Mn‐NC‐mediated mild PTT on bacterial metabolism, MDR *E. coli* were treated by BP@Mn‐NC (25 µg mL^−1^) for 2 h at 37 °C in the supplement of glucose (5.5 mm) and then subjected to 808 nm laser exposure (0.688 W cm^−2^) for 3 min. Bacteria treated by mTP using water bath (45 °C, 3 min) served as the control group. MDR *E. coli* in both groups were further subjected to total RNA extraction, RNA sequencing, and bioinformatic data collection with the assistance of Suzhou PANOMIX Biomedical Tech Co., LTD (China).

### Expression of Bacterial HSPs

To further verify the gene and protein expression levels of bacterial HSPs, MDR *E. coli* were subjected to mTP (45 °C, 3 min) or mild PTT mediated by BP or BP@Mn‐NC (25 µg mL^−1^), followed by subsequent quantitative real‐time polymerase chain reaction (qPCR) and Western blot analyses. For qPCR analysis, treated bacteria were collected using a Bacteria RNA Extraction Kit (R403‐01, Vazyme) for total RNA purification. Thereafter, cDNA was obtained using a Reverse Transcription Kit (A0010, EZBioscience) and qPCR was performed using SYBR Green qPCR Master Mix (A0001, EZBioscience). Relative gene expression was calculated using the 2^−ΔΔCT^ method, and 16S rRNA was used as a reference for normalization. The primer sequences are listed in Table S1 (Supporting Information). For HSPs protein expression detection, the bacterial proteins were extracted from treated bacteria using BeyoLytic Bacterial Active Protein Extraction Reagent (P0013Q, Beyotime). Protein concentration was determined using a BCA Protein Assay Kit (ZJ102, EpiZyme). Equal amounts of protein samples (20 µg) were subjected to SDS‐PAGE and then transferred to a polyvinylidene difluoride membrane. After blocking with 5% (w/v) BSA, the membrane was incubated with primary antibodies at 4 °C overnight and the horseradish peroxidase‐conjugated secondary antibody (1:10000; 115‐035‐003, Jackson ImmunoResearch) at room temperature for 1 h. Immunoreactive bands were visualized using enhanced chemiluminescence reagent (SQ101, EpiZyme) and the semi‐quantitative analysis was conducted using the ImageJ software. Primary antibodies used in this study included anti‐DnaK (1:1000; ab69617, abcam) and anti‐groEL (1:1000; ab82592, abcam).

### Thermal Imaging of Mice Abscess Model

All animal procedures were approved by the Animal Research Committee of Shanghai Sixth People's Hospital Affiliated to Shanghai Jiao Tong University School of Medicine (DWSY2023‐0049). To explore the antibacterial performance of BP@Mn‐NC‐mediated mild PTT in vivo, BALB/c mice (20–22 g, male) were utilized to establish the abscess animal model. MDR *E. coli* suspensions (100 µL, OD = 1) were subcutaneously injected to the left side of the back after dorsal depilation. After bacterial inoculation for 24 h, infected mice were randomly divided into four groups. For the BP + NIR and BP@Mn‐NC + NIR groups, respective dispersions of BP or BP@Mn‐NC (50 µL, 25 µg mL^−1^) were inoculated into the abscesses. For the PBS and PBS + NIR groups, PBS solution (50 µL) was injected as a control. Two hours post these interventions, infected sites of the mice were irradiated by 808 nm laser with an initial power of 0.8 W cm^−2^ and the local temperature was maintained beneath 45 °C by dynamically adjusting the laser power. Thermal images were captured using an infrared thermal imaging camera (FOTRIC, China).

### In Vivo Anti‐Infection Evaluation

After bacterial inoculation, the infected area was monitored every 2 days. Seven days after the treatments, the infected tissues were harvested, photographed, and homogenized in saline for dilution (10^4^ folds), followed by culturing on blood agar plate to assess the In vivo antibacterial properties. In addition, the MDA Content Assay Kit (BC0025, Solarbio) was utilized to examine the MDA generation two days after irradiation exposure in infected tissues from all groups using the tissue homogenates

### Histological Analysis

Eight days after the bacterial inoculation, the infected tissue was collected, fixed in 4% (w/v) paraformaldehyde for 24 h, and paraffin‐embedded. The samples were sliced into 5 µm‐thick sections and processed for H&E and Masson's trichrome staining for histological evaluation. For in vivo ROS detection, the fresh tissue samples collected at the therapeutic day 2 were immersed in optimal cutting temperature compound, quick‐freezed by liquid nitrogen, and cut into 10 µm‐thick sections to process DHE (50102ES02, Yeasen) staining.

### Biosafety Evaluation

Three and eight days after the bacterial inoculation, blood samples were collected in PBS and BP@Mn‐NC + NIR treatment groups for biochemical test. On the 8th day after bacterial inoculation, the mice were sacrificed and major organs including heart, liver, spleen, lung, kidney, and brain were collected in PBS and BP@Mn‐NC + NIR treatment groups and subjected to H&E staining to examine the latent pathological changes. For hemolysis assay, diluted red blood cell suspensions (2%, 0.5 mL) were incubated with gradient concentrations of BP@Mn‐NC (1.25, 2.5, 5, 10, 20, and 40 µg mL^−1^) at 37 °C for 1 h and centrifuged at 1,000 rpm for 10 min, then the absorbance of supernatant at 540 nm was recorded to calculate the hemolysis rate. Triton X‐100 solution (0.5%) and PBS were selected as positive and negative control groups, respectively.

### Statistical Analysis

All experimental data are presented in the form of mean ± standard deviation. Statistical analysis was performed using Student's *t*‐test for binary group comparisons, or one‐way analysis of variance followed by Tukey's *post‐hoc*‐test for multiple group comparisons using GraphPad Prism 8 software (GraphPad Software, USA). Differences were considered significant if *p* < 0.05.

## Conflict of Interest

The authors declare no conflict of interest.

## Supporting information

Supporting Information

## Data Availability

The data that support the findings of this study are available in the Supporting Information of this article.
