# Supplementary material for: Black Phosphorus/MnO2 Nanocomposite Disrupting Bacterial Thermotolerance for Efficient Mild‐Temperature Photothermal Therapy
Source: Adv Sci (Weinh). 2023 Sep 12;10(30):2303911. doi: 10.1002/advs.202303911 (PMC10602513; doi:10.1002/advs.202303911)
Supplement: Supplementary file 1 — Supporting Information [file ADVS-10-2303911-s001.pdf]

ADVANCED SCIENCE
Open Access

Supporting Information

for *Adv. Sci.*, DOI 10.1002/advs.202303911

Black Phosphorus/MnO_2 Nanocomposite Disrupting Bacterial Thermotolerance for Efficient Mild-Temperature Photothermal Therapy

Feng Wang, Qinghe Wu, Guoping Jia, Lingchi Kong, Rongtai Zuo, Kai Feng, Mengfei Hou, Yimin Chai, Jia Xu, Chunfu Zhang* and Qinglin Kang**

Supporting Information

Black Phosphorus/MnO_2 Nanocomposite Disrupting Bacterial Thermotolerance for Efficient Mild-Temperature Photothermal Therapy

Feng Wang[#], Qinghe Wu[#], Guoping Jia, Lingchi Kong, Rongtai Zuo, Kai Feng, Mengfei Hou, Yimin Chai, Jia Xu, Chunfu Zhang*, Qinglin Kang**

[#]These authors contributed equally to this work.

*Corresponding Authors: orthokang@163.com (Qinglin Kang), cfzhang@sjtu.edu.cn (Chunfu Zhang), xujia0117@126.com (Jia Xu).

Affiliations: Department of Orthopedics, Shanghai Sixth People's Hospital Affiliated to Shanghai Jiao Tong University School of Medicine, School of Biomedical Engineering, Shanghai Jiao Tong University, Shanghai 200030, China.

Supplementary Figures

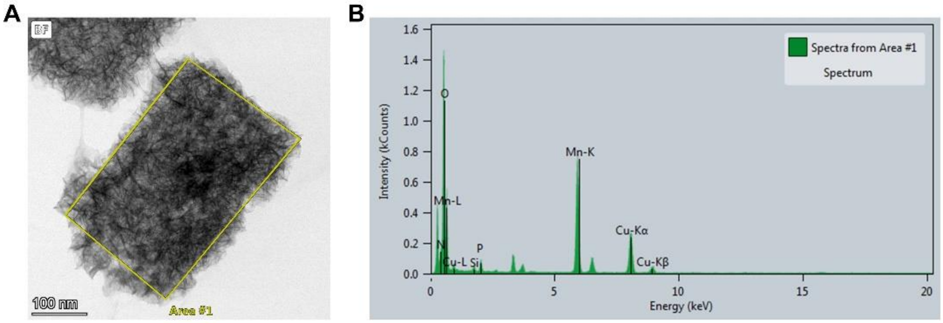

Figure S1. EDS analysis of BP@Mn-NC. (A) TEM image of BP@Mn-NC. The yellow rectangular box indicates the selected area for EDS analysis. (B) EDS spectrum of the selected area in (A).

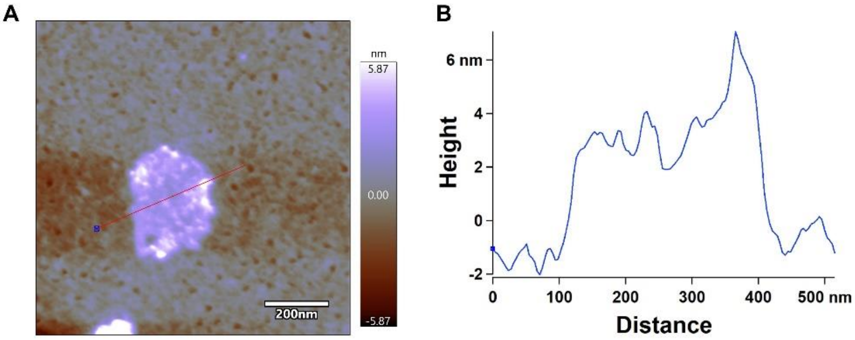

Figure S2. Atomic force microscope image of BP (A) and corresponding line analysis of the height change (B).

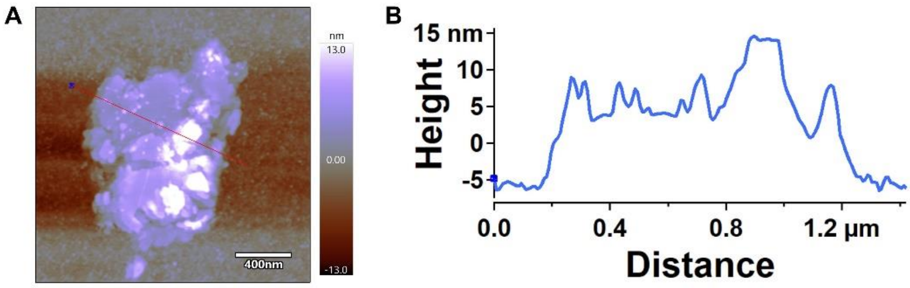

Figure S3. Atomic force microscope image of BP@Mn-NC (A) and corresponding line analysis of the height change (B).

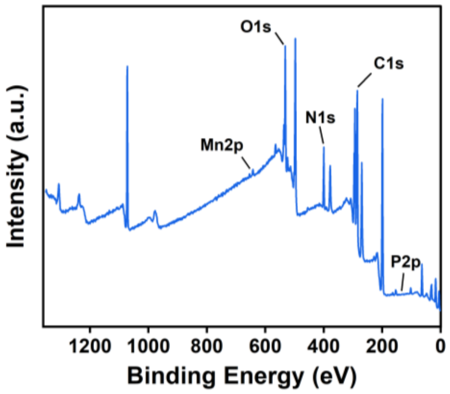

Figure S4. XPS spectrum of BP@Mn-NC.

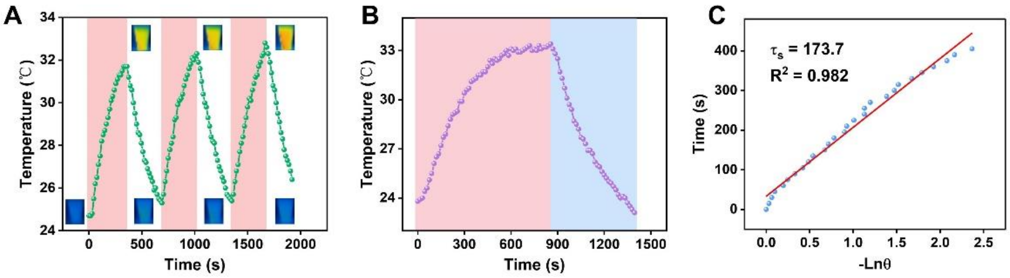

Figure S5. Photothermal conversion capability of BP. (A) Photothermal stability test of BP through three cycles of heating-cooling process. (B) Heating-cooling profile of BP for lasers on/off cycle. (C) Linear cooling time versus -ln(θ) obtained from the cooling period of (B).

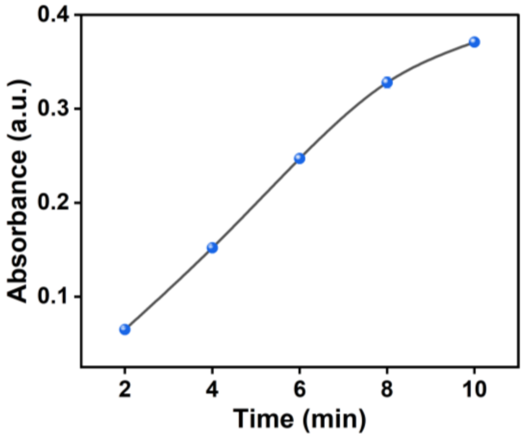

Figure S6. Change of POD-like activity of BP@Mn-NC with reaction time.

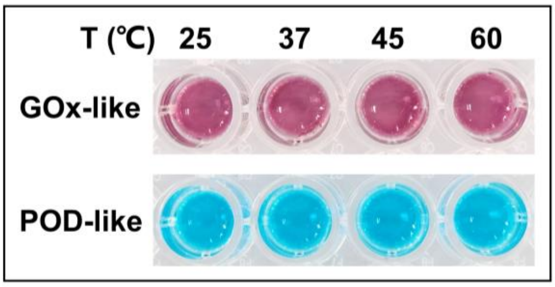

Figure S7. GOx-like and POD-like activity of BP@Mn-NC after treatment at different temperatures.

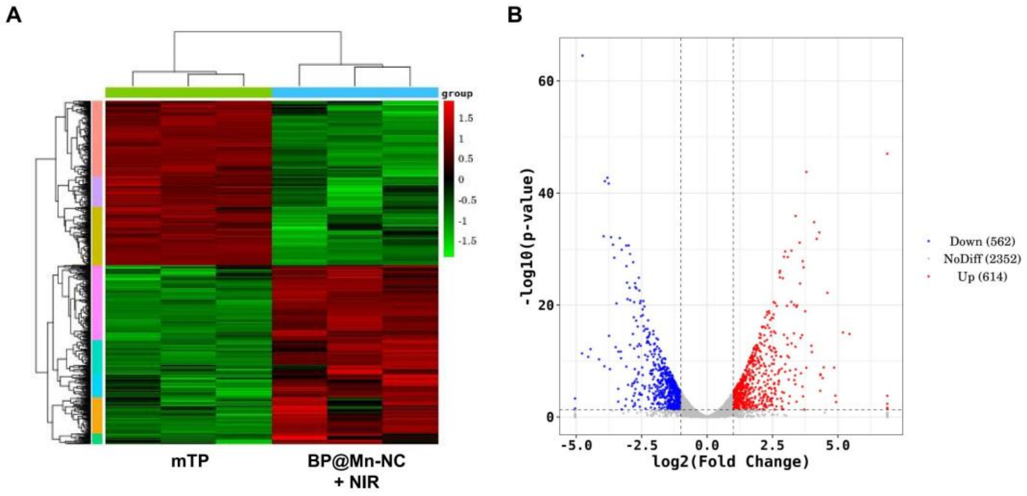

Figure S8. (A) Heatmap and (B) volcano plots of RNA-seq analysis of MDR *E. coli* treated by BP@Mn-NC-mediated (25 μg/mL) mild PTT (808 nm, 0.688 W/cm^2) compared to bacteria treated by mild thermal processing (mTP; 45 °C, 3 min).

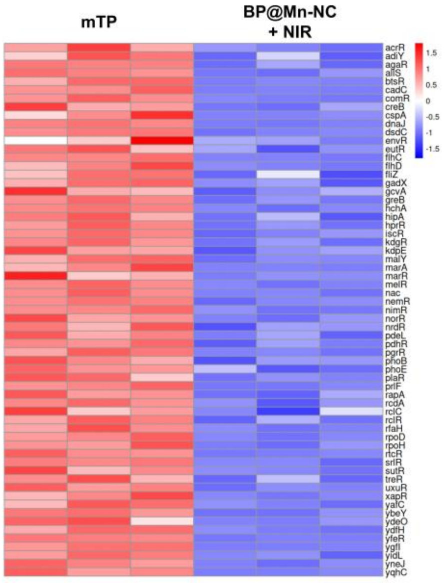

Figure S9. Heatmap of differentially expressed genes involved in bacterial transcriptional activity of MDR *E. coli* in mTP and BP@Mn-NC + NIR groups.

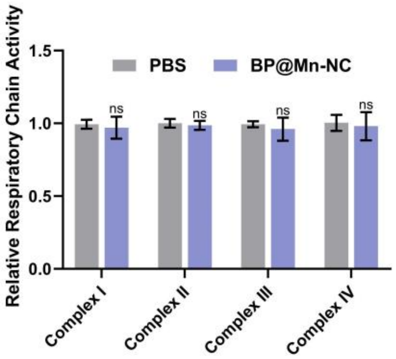

Figure S10. Respiratory chain activity of L929 cells treated by BP@Mn-NC at the antibacterial concentration (25 μg/mL) (n = 3). $^{ns}P > 0.05$.

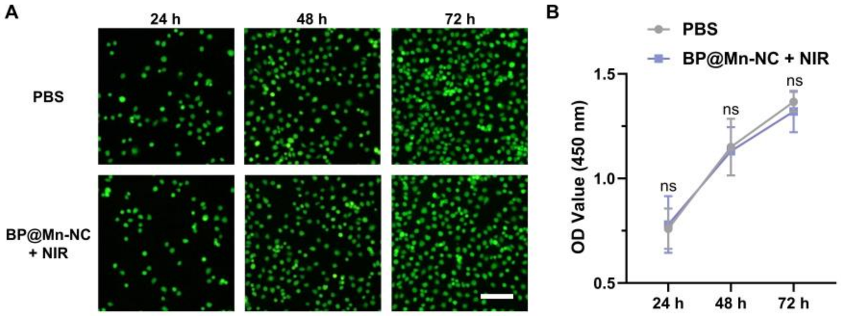

Figure S11. (A) Live/dead staining and (B) CCK-8 experiment on L929 cells treated by BP@Mn-NC-mediated mild PTT (808 nm, 0.688 W/cm^2) at the antibacterial concentration (25 μg/mL) for cell viability detection (n = 3). Scale bar: 200 μm. $^{ns}P >$ 0.05.

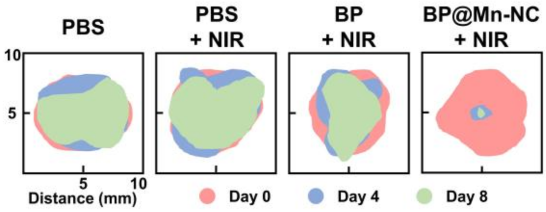

Figure S12. Schematic diagram of the abscess areas from infected mice treated by PBS, BP (50 μL, 25 μg/mL), or BP@Mn-NC (50 μL, 25 μg/mL) followed by NIR irradiation exposure (808 nm, 0.8 W/cm^2).

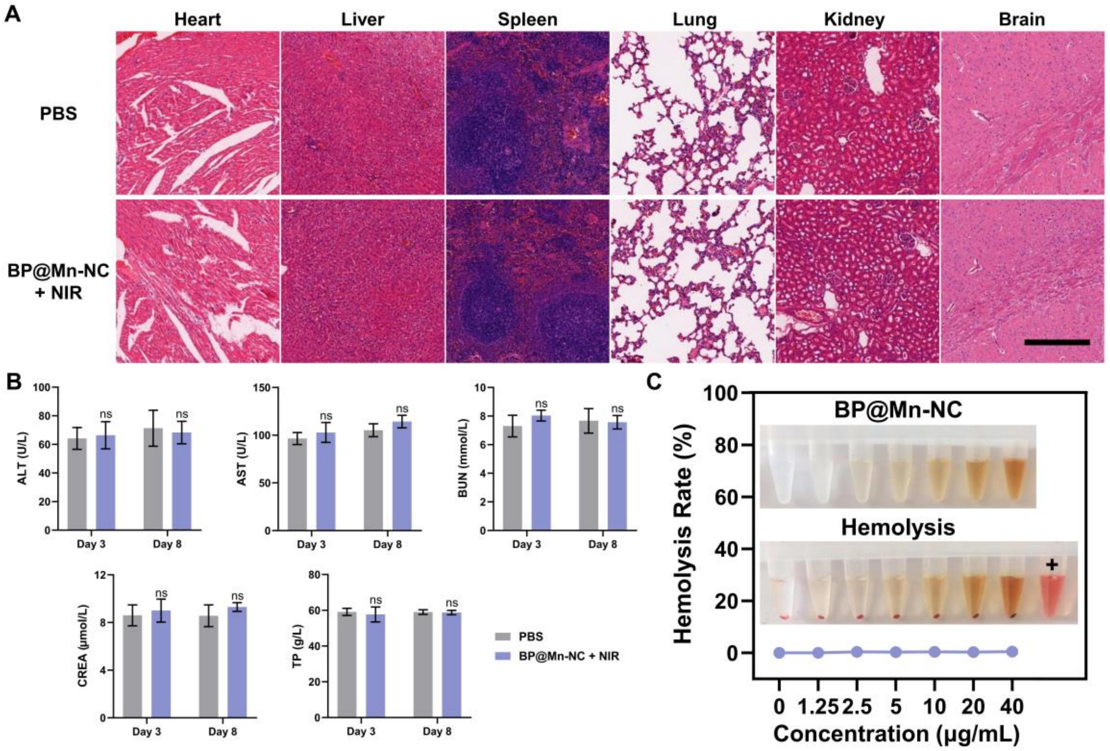

Figure S13. Biosafety evaluation of BP@Mn-NC. (A) H&E staining of main organs (heart, liver, spleen, lung, kidney, and brain) from mice seven days after the treatment of PBS or BP@Mn-NC-mediated (25 μg/mL) mild PTT (808 nm, 0.688 W/cm^2). Scale bar: 300 μm. (B) Blood biochemistry analysis of mice in different groups two and seven days after the treatment (n = 3). (C) Hemolysis rate and corresponding photographs of BP@Mn-NC in gradient concentrations (n = 3). $^{ns}P > 0.05$.

Table S1. Primer sequences for quantitative real-time polymerase chain reaction

Gene	Forward (5’-3’)	Reverse (5’-3’)
DnaK	GTGACAACAAACTTGGCGG TGATG	TCATTGCTTGGCGTGTAGGT TCC
groEL	AAGGTATTGTTGCAGGTGG TGGTAC	CCGCTTCAGTCGTTAAGAAC ATTGC
16S rRNA	GGCCTTGCGCGATTGTATA T	GTGGCGGATCATCTTCTCAG A
